# Frequency of alcohol consumption in humans; the role of metabotropic glutamate receptors and downstream signaling pathways

**DOI:** 10.1038/tp.2015.70

**Published:** 2015-06-23

**Authors:** J L Meyers, M C Salling, L M Almli, A Ratanatharathorn, M Uddin, S Galea, D E Wildman, A E Aiello, B Bradley, K Ressler, K C Koenen

**Affiliations:** 1 Department of Epidemiology, Columbia University, New York, NY, USA; 2 Department of Anesthesiology, Columbia University, New York, NY, USA; 3 Department of Veterans Affairs Medical Center, Mental Health Service Line, Atlanta, GA, USA; 4 Department of Psychology, University of Illinois-Urbana Champaign, Urbana, IL, USA; 5 Institute for Genomic Biology, University of Illinois-Urbana Champaign, Champaign, IL, USA; 6 Department of Molecular and Integrative Physiology, University of Illinois-Urbana Champaign, Urbana, IL, USA; 7 Department of Epidemiology, Gillings School of Global Public Health, University of North Carolina, Chapel Hill, NC, USA; 8 Department of Psychiatry and Behavioral Sciences, Emory University, Atlanta, GA, USA; 9 Howard Hughes Medical Institute, Chevy Chase, MD, USA

## Abstract

Rodent models implicate metabotropic glutamate receptors (mGluRs) and downstream signaling pathways in addictive behaviors through metaplasticity. One way mGluRs can influence synaptic plasticity is by regulating the local translation of AMPA receptor trafficking proteins via eukaryotic elongation factor 2 (*eEF2*). However, genetic variation in this pathway has not been examined with human alcohol use phenotypes. Among a sample of adults living in Detroit, Michigan (Detroit Neighborhood Health Study; *n*=788; 83% African American), 206 genetic variants across the *mGluR*–*eEF2*–AMPAR pathway (including *GRM1, GRM5, HOMER1, HOMER2, EEF2K, MTOR, EIF4E, EEF2, CAMK2A, ARC, GRIA1* and *GRIA4*) were found to predict number of drinking days per month (corrected *P*-value <0.01) when considered as a set (set-based linear regression conducted in PLINK). In addition, a CpG site located in the 3′-untranslated region on the north shore of *EEF2* (cg12255298) was hypermethylated in those who drank more frequently (*P*<0.05). Importantly, the association between several genetic variants within the *mGluR*–*eEF2*–AMPAR pathway and alcohol use behavior (i.e., consumption and alcohol-related problems) replicated in the Grady Trauma Project (GTP), an independent sample of adults living in Atlanta, Georgia (*n*=1034; 95% African American), including individual variants in *GRM1, GRM5, EEF2*, *MTOR*, *GRIA1, GRIA4* and *HOMER2* (*P*<0.05). Gene-based analyses conducted in the GTP indicated that *GRM1* (empirical *P*<0.05) and *EEF2* (empirical *P*<0.01) withstood multiple test corrections and predicted increased alcohol consumption and related problems. In conclusion, insights from rodent studies enabled the identification of novel human alcohol candidate genes within the *mGluR*–*eEF2*–AMPAR pathway.

## Introduction

Advances in genomic technology have enabled progress toward identifying genes involved in alcohol use disorders. This has led to a growing recognition of the need to understand the mechanisms underlying genotype–phenotype associations for continued progress in the translational sciences.^1, 2, 3^ The preference for some rodents to drink alcohol while others avoid alcohol has been used as a tool to study and target specific biological underpinnings of alcohol use behaviors since the 1940s.^4^ Recently, reviews of the state of the science for animal models and human association studies in alcohol use disorders have concluded that alcohol consumption is an important area of research where consilience between human and animal studies is possible. In this respect, the wealth of potential therapeutic targets collected from validated animal models of alcohol drinking could contribute to advances in the identification of mechanisms underlying genotype–phenotype associations in humans.^5, 6^

Chronic alcohol abuse is thought to lead to adaptations of the glutamate system that result in a ‘hyper-glutamatergic' state.^7^ Metabotropic glutamate receptors (mGluRs) are G-protein-coupled receptors that are expressed throughout the central nervous system that can be found in both the pre- and postsynaptic membrane of glutamatergic synapses. MGluRs and downstream signaling pathways are known to regulate several alcohol-related behaviors in animal studies. For example, pharmacological inhibition and genetic targeting of Group 1 mGluRs, mGlu1 and 5 *(GRM1, GRM5)* significantly reduces alcohol self-administration and relapse-like behavior in rodents.^8, 9, 10, 11, 12, 13, 14, 15^ Furthermore, downstream pathways including signaling through the mGlu1/5 scaffolding proteins and downstream protein kinases influence excessive alcohol consumption.^16, 17, 18, 19, 20, 21, 22^ As a result of this compelling data, mGluRs are currently being explored as biomarkers for alcohol abuse and as pharmacologic targets for therapeutic treatment of alcohol use disorders.^23, 24^

**Figure 1:**
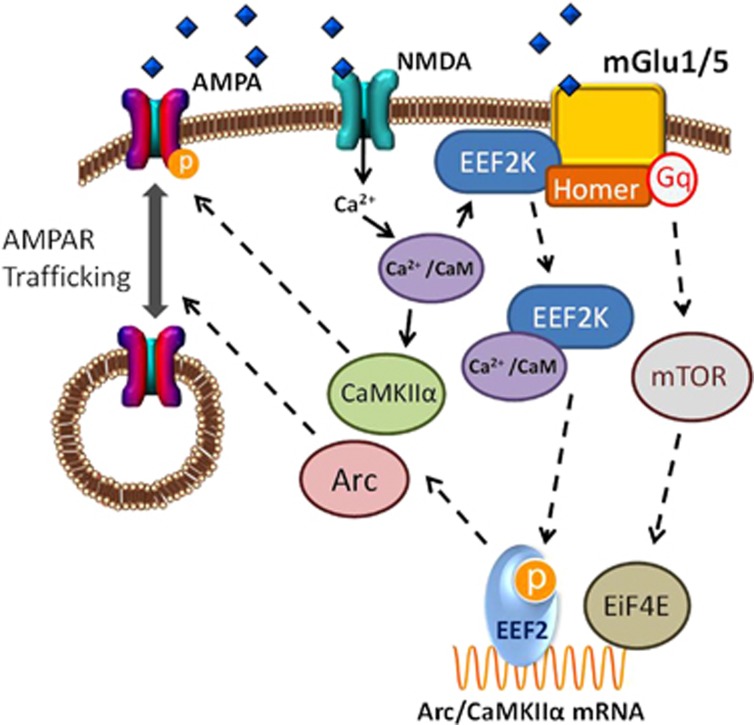
Simplified schema demonstrating proposed *mGluR*–*eEF2*–AMPAR pathway: Postsynaptic binding of glutamate leads to activation of ionotropic (AMPAR, NMDAR shown) and metabatropic (mGlu1/5 shown) glutamate receptors. Activation of mGlu1/5 leads to Gq activation of PLC (not shown) and the mTOR pathway that can affect the activity of Ei4FE and initiation of local RNA translation. Binding of Ca^2+^/CaM to EEF2K releases eEF2K-Ca/CaM complex from mGlu1/5 and scaffolding protein Homer. EEF2K-CaM phosphorylates EEF2 and switches its activity in global RNA translation to specific RNA translation including CaMK2α and Arc. CaMK2α and Arc interact with AMPARs and with downstream proteins to modulate trafficking of AMPARs in the postsynaptic membrane and regulate synaptic plasticity. AMPAR, α-amino-3-hydroxy-methyl-4-isoxasolepropionic acid receptor; CaMK2α, calcium/calmodulin-dependent protein kinase II alpha; mTOR, mechanistic target of rapamycin.

Alterations in synaptic strength are one potential physiological mechanism underlying the maladaptive learning processes associated with addiction.^25, 26^ One particular Group 1 mGluR (mGlu1 and 5) pathway that has gained interest in addictive disorders is the mechanism by which mGluR activation controls rapid local translation of proteins involved in long-term synaptic plasticity,^27^ which can occur through the phosphorylation of eukaryotic translation elongation factor 2 (eEF2; Figure 1).^28, 29^ Several upstream scaffolding (i.e., Homer homolog 1-2 (HOMER1-2)), and signaling proteins (i.e., mechanistic target of rapamycin (MTOR), and *eEF2*-kinase (eEF2K)), influence eEF2 to act as a switch from promoting global protein synthesis to the local translation of a set of proteins, such as calcium/calmodulin-dependent protein kinase II alpha, (CaMK2α) and activity-related cytoskeleton protein Arc (ARC). These proteins (CaMK2α, ARC) regulate the trafficking of α-amino-3-hydroxy-methyl-4-isoxasolepropionic acid receptors (AMPA) glutamate receptors (i.e., GRIA1-4), thereby affecting synaptic strength. Further, AMPA receptors (AMPARs), ARC and CaMK2α have independently been implicated in alcohol consumption in rodent models.^30, 31, 32^ Recent evidence has shown that regulating AMPAR trafficking through mGluRs in specific neural circuits can reverse addictive behaviors in rodents.^33, 34^ There is an abundance of evidence to suggest a link between variants in the *mGluR*–*eEF2*–AMPAR pathway and alcohol use behaviors; however, this pathway has not previously been examined in humans. Therefore, the primary goal of this study is to examine genotypic variation in the *mGluR*–*eEF2*–AMPAR pathway as it relates to alcohol use behavior in humans.

Although there is a large literature examining genetic influences on alcohol use behaviors, comparatively fewer studies have examined epigenetic profiles in alcohol consumption. Epigenetic modifications are regulatory mechanisms that alter gene expression without changing DNA sequence. One of the best-studied epigenetic mechanisms is DNA methylation, which may change activity in the promoter region, as well as other genic regions, and thus regulate gene transcription. Accumulating data indicate that heavy alcohol consumption can alter the methylation status of specific genes. One of the first studies^35^ showed a higher peripheral blood DNA methylation level in the α-synuclein gene (*SNCA*) in alcoholic patients than in controls. Since that time, subsequent studies using peripheral blood indicated that several other genes^36, 37, 38^ were more highly methylated in subjects with alcoholism than in controls. Only one of these studies examined methylation effects in an African American sample, and found that CpG sites in multiple genes were hypermethylated in alcoholic cases,^39^ which is consistent with findings in other populations.^36, 37, 38^

Drawing on samples from two community-based majority African American cohorts, the Detroit Neighborhood Health Study (DNHS) and the Grady Trauma Project (GTP), we assessed the association between frequency of alcohol consumption and genotypic variants in the *mGluR*–*eEF2*–AMPAR using gene-based and pathway-based analyses.^40^ Given the likely multifactorial nature of alcohol consumption, we hypothesize that genetic variants in the *mGluR*–*eEF2*–AMPAR pathway will have a combined effect on risk for heavy alcohol consumption and related problems (in addition to other genetic and environmental influences). In the DNHS, we explore one potential mechanism underlying the genotype–phenotype association, by testing the association between alcohol consumption and methylation sites in the *eEF2* region, a functional target of the *mGluR*–*eEF2*–AMPAR pathway.

## Materials and methods

### The DNHS

Participants include 788 individuals with available genetic data who participated in the DNHS, a longitudinal population-based cohort of male and female (57.7%) adults (18+ mean age: 52.7, s.d.: 16.4) living in Detroit, MI, USA. Participants were administered a 40-min assessment, which included demographic variables including self-identified race, sex, age, as well as questions on alcohol use, and other behavioral characteristics. Assessments were administered using structured interviews, and each participant received $25 for their participation in the survey. Written and verbal informed consent was obtained for all the participants, and all procedures in this study were approved by the institutional review boards of the University of Michigan. When asked how they would describe their racial background, 82.5% endorsed ‘Black/African American', with the remainder of the sample endorsing ‘Asian', ‘American Indian', or ‘Alaska Native', paralleling census-based estimates on individuals living in Detroit, MI, USA. Further details regarding the DNHS study background and collection can be found in Uddin *et al.*^41^

### Frequency of alcohol consumption

The following question was used to assess alcohol consumption: ‘Thinking about the past 30 days on how many days did you drink any alcoholic beverages?' Only individuals who had evidence of alcohol exposure were included in analyses so that genetic and environmental influences on the decision to initiate alcohol drinking are not confounded with genetic and environmental influences on current frequency of consumption.^42^ 35.7% of the full sample had never consumed alcohol in their lifetime and were excluded from further analyses.

### Genotypic data (DNHS)

Respondents were also asked to provide blood specimens by way of venipuncture (or by way of saliva when blood was unavailable, *n*=125), and received an additional $25 if they elected to do so. A total 802 samples were collected from consenting participants for genetic analysis. DNA samples were sent to the Applied Genomics Technology Facility (Wayne State University, Detroit, MI, USA) for genotyping using the HumanOmniExpress BeadChips (Illumina, San Diego, CA, USA). Further details regarding genome-wide association procedures have been published previously.^43^ Samples were removed due to low call rate (<95%) and duplicate issues, with a remaining sample of 795 individuals (418 women and 367 men). A total of 688 890 single-nucleotide polymorphisms (SNPs) passed quality control filters (call rate >95%, minor allele frequency >0.01, Hardy–Weinberg disequilibrium *P*>1 × 10^−^^6^).

### Population stratification

We used multi-dimensional scaling (MDS) analysis of genome-wide identity-by-state data implemented in PLINK version 1.07 (ref. 44) to determine genetic ancestry in the whole sample. The analysis was conducted using the 688 890 SNPs that passed quality control filters previously described. Of the 31 dimensions identified from this MDS analysis, the first two components identified clusters that highly correlate with African American and European American self-report racial identification. To reduce population stratification, we removed all individuals who described themselves as ‘White/Caucasian' in the association analyses (*n*=7). All 31 MDS components were used to adjust for any remaining population stratification in the association analyses.

In the following analyses, we analyzed 206 SNPs available and meeting quality control criteria (call rate >95%, minor allele frequency >0.01, Hardy–Weinberg disequilibrium *P*>1 × 10^−6^) within 100 kb upstream and downstream of each of the genes of interest in the *mGluR*–*eEF2*–AMPAR pathway (detailed in Table 1 and Figure 1), which was compiled from several lines of research demonstrating the role of Group 1 mGluRs and downstream signaling pathways in the local translation of proteins in neuronal dendrites.^45, 46, 47^ We focused on genes encoding proteins that initiate the local translation of *CaMK2α* and *ARC* with *eEF2*. AMPA receptor genes (*GRIA1 and 4*) were included as the trafficking of these proteins are the functional consequence targeted by this pathway. Genotypic analyses included 15 SNPs in glutamate receptor metabotropic 1 (*GRM1*), 38 SNPs in glutamate receptor metabotropic 5 (*GRM5*), 31 SNPs in Homer homolog 1 (*HOMER1*), 38 SNPs in Homer homolog 2 (*HOMER2*), 15 SNPs in *MTOR*, 19 SNPs in eukaryotic elongation factor 2 kinase (*EEF2K*), 8 SNPs in Eukaryotic Translation Initiation Factor 4E (*EIF4E*), 4 SNPs in Eukaryotic Translation Elongation Factor 2 (*EEF2*), 13 SNPs in calcium/calmodulin-dependent protein kinase II (*CAMK2A*), 3 SNPs in activity-related cytoskeleton protein Arc (*ARC*), 9 SNPs in glutamate receptor, ionotropic, AMPA 1 (*GRIA1*) and 13 SNPs in glutamate receptor, ionotropic, AMPA 4 (*GRIA4*). Note that *GRIA2* and *GRIA3* were excluded from these analyses due to exclusions based on quality control criteria (call rate >95%, minor allele frequency >0.01, Hardy–Weinberg disequilibrium *P*>1 × 10^−6^).

### DNA methylation data (DNHS)

A total 179 blood samples were obtained from consenting participants via venipuncture and whole-blood-derived genomic DNA was isolated from 400 ml of whole blood using the DNA Mini Kit (Qiagen, Valencia, CA, USA) following the manufacturer's recommended protocol. One microgram of the resulting genomic DNA was then submitted for DNA methylation profiling by the genomics core facility, AGTC, at Wayne State University using the HumanMethylation450 BeadChip (Illumina). Genomic DNA was denatured and bisulfite converted using the Zymo EZ-96 DNA Methylation kit deep well format (Zymo Research, Irvine, CA, USA), following the alternative incubation protocol. Bisulfite converted DNA was amplified, fragmented and hybridized to the HumanMethylation450 Beadchip using standard Illumina Infinium Methylation HD protocol. Arrays were then scanned on the Illumina iScan using standard Illumina scanner settings. The resulting raw DNA methylation data were imported from IDAT files, Illumina's proprietary file format, using the R package ChAMP^48^ of Bioconductor.^49^ Use of over-the-counter and prescription drug medication was documented (0=no medication use; 1=any medication use) during the in-home visits when the biologic specimens were collected.

In accordance with this study's hypothesis, and to reduce the number of tests run (and the related need for multiple test corrections), we analyzed eight CpG sites available within 100 kb upstream and downstream of eukaryotic translation elongation factor 2 (*EEF2*) detailed in Table 2. All individuals who had data at the eight CpG sites and alcohol consumption data were included in analyses (analytic sample=154).

### The GTP, genotypic replication sample

The GTP is a population of male and female (71.8%) adults (Mean age: 39.5, s.d.: 13.4) approached while in the waiting rooms of primary care or obstetrical–gynecological clinics of Grady Memorial Hospital in Atlanta, GA, USA. This population predominantly (95.2%) self-identifies as ‘African American/Black', the remainder of the sample self-identifies as ‘White' (3.3%) or ‘Other.' Participants were paid $15 for this phase of the study. Written and verbal informed consent was obtained for all participants, and all procedures in this study were approved by the institutional review boards of Emory University School of Medicine and Grady Memorial Hospital, Atlanta, GA, USA. Participants completed a battery of self-report measures that took 45 to 75 min to complete.

### Alcohol consumption and related hazardous behaviors

Measures of alcohol use were obtained by verbal interview using the Alcohol Use Disorders Identification Test (AUDIT^50^). Each of the 10 AUDIT items is rated on a scale of 0–4, with higher scores reflecting more problematic alcohol drinking. The first question of the AUDIT was used to examine alcohol consumption: ‘During the last year, on average, how many drinks containing alcohol do you have on a typical drinking day?' Individuals who had never consumed alcohol in their lifetime (43%, those answering ‘never' to the question above) were excluded from analyses. Other AUDIT items address problematic behavior or consequences accompanying heavy alcohol consumption, such as ‘How often during the last year have you failed to do what was normally expected of you because of drinking?' and ‘How often during the last year have you been unable to remember what happened the night before because of drinking?'. Thus, the GTP measure of alcohol consumption differs from that obtained in the DNHS in its time period (DNHS: past 30 days; GTP: past year) and that it also includes alcohol consumption-related hazardous behaviors. Twin and molecular genetic studies have shown that there is evidence for shared genetic influences across different phenotypes.^51, 52^ Therefore, convergent findings from these two alcohol phenotypes (frequency of consumption in past 30 days vs AUDIT scores) would increase support for findings. Full data were available on 1034 participants.

### Genotypic data (GTP)

GTP participants provided a saliva sample using Oragene saliva kits (DNAGenotek, Ottawa, ON, Canada), and genotyping was conducted using Illumina's Omni1-Quad BeadChip. The Omni1-Quad BeadChip interrogates 1 011 219 individual SNPs. Genotypes were called using Illumina's GenomeStudio software. PLINK version 1.07 (ref. 44) was used to perform quality control analyses on the genotypic data. When comparing the genotyped individuals to the non-genotyped individuals, no significant differences in demographic or phenotypic measures relevant to this study were found.

### Population stratification

MDS analysis conducted in PLINK was run on 1 011 219 SNPs to infer axes of ancestry. On the basis of MDS, African American subjects who fell within three standard deviations of the medians of the first and second MDS component in this sample were retained (92%). To reduce population stratification, we removed all individuals who described themselves as ‘White' in the association analyses (3.3%). All MDS components were used to adjust for any remaining population stratification in the association analyses.

### Statistical analyses

For the DNHS samples (genotypic *n*=676; methylation *n*=154), descriptive statistics on key variables, as well as comparisons between the subset of individuals with methylation data to the genotypic sample are provided in Table 3. In addition, descriptive statistics on key variables for the GTP genotypic sample are provided in Table 3. Frequency of alcohol consumption was standardized (*z*-scored) and modeled as a continuous variable. We assessed main effects of all 206 SNPs and eight CpG sites available using the SNP or methylation beta value as predictors, controlling for demographic characteristics (age, sex and ancestry components), behavioral characteristics (smoking, medication use for methylation analyses only) and peripheral blood mononuclear cell count. Methylation beta values were centered to the mean in all models.

Gene-based and pathway-based tests were conducted in PLINK (Purcell, 2003) using set-based analyses (‘—set-test'). Gene-based tests included all available SNPs in a given gene, corrected for the number of independent signals (i.e., linkage disequilibrium blocks) within that gene set. Pathway-based tests included all available SNPs within each of the genes considered in the *mGluR*–*eEf2*–AMPAR pathway, corrected for the number of independent signals (i.e., linkage disequilibrium blocks) within that pathway set. In the main effects model, SNP coefficients were accepted as significant if the 100 000 permutations of the set-based regression analysis (Bonferroni corrected for the number of independent signals within the set) produced an empirical *P*-value <0.05. Coefficients for gene methylation value were accepted as significant if *P*<0.006, based on the Bonferroni correction for multiple testing (*P*-value significance threshold of 0.05/8 CpG sites examined was *P*<0.006). Finally, *post hoc* analyses of individual SNPs included in the *mGluR*–*eEf2*–AMPAR pathway were conducted to provide further information on each individual variant. These analyses were conducted in PLINK using linear regression for a quantitative phenotype.

In an effort to replicate the genotypic analyses conducted in the DNHS, parallel analyses were conducted in the GTP (genotypic *n*=1352). In the GTP, AUDIT scores were modeled as continuous variables. Using the same parameters as described above (regression analyses controlling for demographic characteristics: age, sex and principal components), gene-based and pathway-based tests were conducted in PLINK using set-based analyses. As described above, *post hoc* analyses of individual SNPs included in the *mGluR*–*eEf2*–AMPAR pathway were conducted to provide further information on each individual variant in the GTP.

## Results

Key demographic characteristics of the DNHS and GTP participants are presented in Table 3.

### Genotypic results (gene-based and pathway-based set tests)

Pathway-based and gene-based tests of association are detailed in Table 1, and individual SNP associations are provided in Supplementary Table S1.

Gene-based analyses indicated that *GRM1* (empirical *P*<0.05), *MTOR* (empirical *P*<0.05), *EIF4E* (empirical *P*<0.05), *EEF2* (empirical *P*<0.05) and *CAMK2A* (empirical *P*<0.05) withstood multiple test corrections and predicted increased alcohol consumption in the DNHS. Pathway-based analyses indicated that 206 variants across each of these genes considered as a single set significantly predicted alcohol consumption in the DNHS (empirical *P*<0.0001). This association withstood correction for the number of independent signals tested across these 206 variants (seven linkage disequilibrium blocks). Several individual SNPs across *GRM1, GRM5, HOMER1, HOMER2, MTOR, EIF4E, EEF2, CAMK2A* and *GRIA4* were associated with increased alcohol consumption in the DNHS (Supplementary Table S1). However, most of these SNP associations did not remain significant after a Bonferroni test correction (0.05/206 individual variants requires a *P*-value threshold of *P*<0.0002).

In an effort to replicate our findings from the DNHS, we tested for association between variants in *GRM1, GRM5, HOMER1, HOMER2, EEF2K, MTOR, EIF4E, EEF2, CAMK2A, ARC, GRIA1, GRIA4* and alcohol use behavior (AUDIT) in the GTP. Although the association between this pathway (174 SNPs) and alcohol consumption only trended towards significance in the GTP (pathway-based empirical *P*-value=0.09), several variants within this pathway were associated with alcohol consumption in the GTP, including variants in *GRM1, GRM5, HOMER4*, *MTOR, EEF2, GRIA* and *GRIA4* (*P*<0.05; individual SNP results detailed in Supplementary Table S1). Further, gene-based analyses indicated that *GRM1* (empirical *P*<0.05), *and EEF2* (empirical *P*<0.05), withstood multiple test corrections and, as in the DNHS, predicted increased alcohol use behavior in the GTP (Table 1).

### DNA methylation results

**Figure 2:**
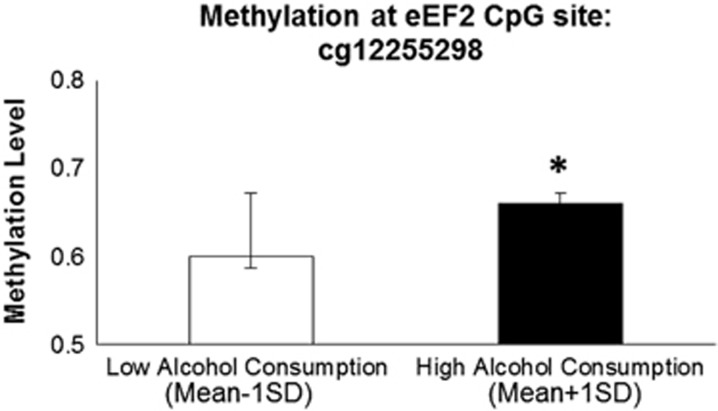
Increased methylation of cg12255298 (located in the 3′-UTR of EEF2 on Chr 19) for individuals with higher drinking days per month in the DNHS. Although alcohol consumption was analyzed continuously, it is presented dichotomously here for ease of presentation. Low alcohol consumption is defined as 1 s.d. below the mean value of alcohol consumption in the DNHS, whereas high alcohol consumption is defined as 1 s.d. above the mean value of alcohol consumption in the DNHS. Significant difference, *P<0.05. DNHS, Detroit Neighborhood Health Study; UTR, untranslated region.

Of the eight CpG sites examined across the *EEF2* region in the DNHS, one site (cg12255298), located in the 3'-untranslated region on the north shore of eEF2 (chromosome 19, BP: 3 976 193–3 976 193), was significantly and positively related to alcohol consumption (*B*=0.419, *P*=0.004) and withstood a multiple test correction (0.05/8=0.006). For individuals with higher drinking days per month (1 s.d. above the mean), cg12255298 was hypermethylated as compared with individuals with fewer drinking days per month (1 s.d. below the mean). This relationship is depicted in Figure 2.

## Discussion

We believe this is the first study to examine the association between human alcohol use and variants in the *mGluR*–*eEf2*–AMPAR pathway (Figure 1), which have been implicated in rodent studies of alcohol consumption and addiction. Drawing on samples from a community-based majority African American cohort, the DNHS, we found that genetic variants within this pathway (including *GRM1, GRM5, MTOR, EIF4E, EEF2* and *CAMK2A*) predicted number of drinking days per month when considered as a set. Further, a CpG site in the 3′-untranslated region on the north shore of *EEF2* (cg12255298) was hypermethylated in those who drank more frequently. Importantly, the association between alcohol use behavior and several SNPs within this pathway (including SNPS within *GRM1, HOMER1, HOMER4, EIF4E, EEF2, GRIA1* and *GRIA4*) were replicated in an independent sample, the GTP. When considered as a set in the GTP, the *mGluR*–*eEf2*–AMPAR pathway only approached significance (empirical *P*-value=0.09), with significant gene-based effects observed for *GRM1* and *EEF2*. Taken together, these results suggest that insights gained from rodent studies enabled the examination of novel alcohol candidate genes within the *mGluR*–*eEf2*–AMPAR pathway. Such pathway-based approaches,^40^ combined with functional genomic data, can aid the field in identifying mechanisms underlying phenotype–genotype associations.

Several studies have linked *mGluR*–*eEF2*–AMPAR related genes (for example, *GRM1, GRM5, CaMK2α, GRIA1-4*) to alcohol use behaviors in rodents.^9, 10, 11, 17, 18, 19, 20, 21, 22, 53, 54, 55, 56, 57, 58^ However, relatively few studies have examined these genetic variants in human genetic association studies. Exceptions include variants in *GRM5, CAMK2A* and *GRIA2*, which have independently been identified in association studies of alcohol use phenotypes in humans,^31, 40, 59^ with preclinical rodent models supporting their role in functional regulation of alcohol self-administration.^8, 19, 22, 31, 32, 60, 61^ To our knowledge, the *mGluR*–*eEF2*–AMPAR pathway variants implicated in this study have not been previously associated (at the genome-wide level) with alcohol consumption behavior in large genome-wide association studies (GWAS). This includes the largest GWAS of alcohol consumption behavior conducted on 26 316 individuals (replication *N*=21 185; ref. 59). However, one recent study^31^ used GWAS data to test 23 SNPs within *CAMK2A*; 7/23 SNPs were found to be significantly associated with alcohol dependence, one of which (intronic *CAMK2A* SNP rs7711562) is implicated in this study's findings. Notably, the participants in the Schumann *et al.* GWAS, as well as other previously published large GWAS of alcohol use behavior (for example, findings from the Collaborative Study on the Genetics of Alcoholism^62^ and IMAGEN^59^) are largely of European ancestry. This study's findings may indicate the potential to identify novel genetic risk variants for alcohol use behavior in populations of African ancestry. Importantly, these findings should be replicated in other admixed populations to determine whether *mGluR*–*eEF2*–AMPAR effects are more relevant to alcohol use behavior in specific populations.

Although this study emphasizes a systems-based approach to genetic association (i.e., gene-based and pathway-based tests of association), the individual SNPs most robustly associated with alcohol use behavior in this study are detailed in Supplementary Table 1. Little is known about the functional relevance of most of these SNPs, however we do note that *GRM1* variant rs2235875, which is significantly associated with alcohol use behavior across the DNHS and GTP, is an intronic variant and rs3170368, which is also associated with alcohol use behavior across both samples, is a variant located in the 3′-untranslated region of *EEF2*.

Previous studies examining methylation patterns in alcohol use phenotypes have found that CpG sites are typically hypermethylated among alcoholic cases as compared with controls.^35, 36, 37, 39^ The methylation patterns observed in this study are consistent with previous studies of other genes,^35, 36, 37, 39^ in that CpG sites located within *EEF2* are hypermethylated in heavier drinkers (1 s.d. above the mean level of alcohol consumption) as compared with lighter drinkers (1 s.d. below the mean level of alcohol consumption). Given the relatively small sample sizes in the present study, particularly for the epigenetic subsample, these genetic and epigenetic effects should be replicated in future studies with greater sample size and ancestral diversity.

Several mechanisms linking altered excitatory neuroplasticity to substance abuse have been hypothesized.^23, 63^ For example, there has been an increased focus on how drugs of abuse (including alcohol) affect glutamate synaptic plasticity in mesocorticolimbic circuitry that may explain the selective and enduring nature of addiction.^64^ It is now well supported that acute and long-term alcohol exposure alters glutamatergic synaptic plasticity in preclinical animal models.^56, 65, 66, 67, 68^ Signaling through mGluRs can modulate synaptic plasticity by regulating the insertion of AMPARs in the postsynaptic membrane. AMPAR trafficking underlies multiple mechanisms of neuroplasticity implicated in drug addiction including unsilencing of synapses, long-term potentiation, long-term depression and synaptic scaling.^27, 45, 69^ Genotypic variation in this pathway may lead to individual differences in the response to alcohol consumption. For instance, SNPs detected here may influence the expression of proteins in this pathway and evidence from genetic manipulations in preclinical models support their role in alcohol consumption.^15, 16, 17, 18, 20, 21, 55^ Furthermore, long-term alterations in glutamate transmission that occur following drug/alcohol exposure have subsequently been shown to functionally regulate addictive behaviors such as drug sensitization, motivation and relapse-like behavior.^70, 71, 72^ Therefore, the variants in the *mGluR*–*eEF2*–AMPAR pathway may not only influence behavioral components of alcohol consumption but also the susceptibility to developing an alcohol use disorder. This has led to the interest in AMPAR and mGluR modulators as potential pharmacotherapies for alcohol use disorders.^23, 24, 63, 64^ Given that *GRM1* (and not *GRM5*) was found to be associated with alcohol use behavior, these findings emphasize its role regulating alcohol consumption in humans.

This study indicates a role for the *mGluR*–*eEF2*–AMPAR pathway in human alcohol use behavior, and adds support to the cross-species evidence for this pathway in alcohol use behavior. Furthermore, this study is the first to find a significant association of *GRM1* and/or *EEF2* with alcohol use behavior. However, the results of this study should be interpreted in light of several key limitations. First, sample sizes in both the DNHS and the GTP were limited; this study only had adequate power to detect 78% and 92% of main effects with risk ratios >1, given the parameters of the DNHS and GTP, respectively. However, the cross-species consilience, as well as the replication of variants (*GRM1* and *EEF2*) in an independent sample lessens this concern.^5, 6^ Second, the present analysis uses data available at a single time point, using retrospective reports of alcohol behavior measured differently in two population-based samples. Twin and molecular genetic studies have shown that different measures of alcohol phenotypes can yield significantly different findings.^51, 52^ However, there is also evidence for shared genetic influences across different phenotypes.^51^ Therefore, convergent findings from these two alcohol phenotypes (frequency of consumption in past 30 days vs AUDIT scores) increase support for these findings. In addition, previous studies have found that individuals typically underreport their drinking consumption,^73, 74^ which would likely diminish the association effects observed in this study. However, recent studies directly comparing retrospective reports to observational data conclude that this bias is significantly less pronounced in regular drinkers and when less time has passed since the drinking days being recalled.^74^ Therefore, the past recent drinking period examined in this study somewhat mitigates this concern. Third, the variants included in this pathway analysis were limited by availability of genotypes in the DNHS and GTP. For example, *GRIA2* and *GRIA3* were excluded from this study due to exclusions based on quality control criteria (call rate >95%, minor allele frequency >0.01, Hardy–Weinberg disequilibrium *P*>1 × 10^−^^6^). Finally, in addition to genetic and epigenetic influences on alcohol consumption, key aspects of the contextual environment have a large impact on alcohol use behaviors. Due to the focus on a novel pathway, and limited power to examine gene–environment interactions in this statistical paradigm, environmental influences were not examined in the present study and should be included in future studies.

Although advances in genomic technology and bioinformatics have enabled progress toward identifying some genes involved in alcohol use disorders, few studies conducted in human samples have identified mechanisms underlying genotype–phenotype associations. These mechanisms are important for downstream use in the translational sciences including pharmacologic, clinical and public health interventions.^1, 2, 3^ In this study, we utilized two established approaches to delve further into the associations observed between genotypic variation and alcohol consumption in two human samples. This study used insights gained from rodent studies to identify novel alcohol candidate genes within the *mGluR*–*eEf2*–AMPAR pathway and to elucidate mechanisms related to altered glutamatergic transmission and synaptic plasticity. Given the findings from the present study, there is growing support for the role of *GRM1* and *EEF2* in alcohol use behaviors across species and across methodologies. Such translationally informed pathway approaches offer promise in propelling the field towards understanding mechanisms underlying phenotype–genotype associations.

## Figures and Tables

**Table 1:** *MGluR*–*eEf2*–AMPAR pathway predicting alcohol use behavior in the DNHS and GTP

*Gene/pathway*	*Number of SNPs*	*LD blocks*	*Empirical* P*-value*
*Detroit Neighborhood Health Study (outcome: drinking days per month)*			
*Gene-based sets*			
* * *GRM1*	15	3	0.009
* * *EIF4E*	8	2	0.019
* * *MTOR*	15	3	0.039
* * *CAMK2A*	13	6	0.039
* * *EEF2*	4	1	0.050
* * *HOMER1*	31	2	0.128
* * *GRIA1*	9	2	0.198
* * *EEF2K*	19	4	0.336
* * *HOMER2*	38	5	0.366
* * *GRIA4*	13	2	0.386
* * *GRM5*	38	2	0.505
* * *ARC*	3	1	0.755
			
*Pathway-based set*			
*** * ***MGluR*–*eEf2*–AMPAR	206	7	0.009
			
*Grady Trauma Project (outcome: AUDIT score)*			
*Gene-based sets*			
* * *GRM1*	14	2	0.052
* * *EIF4E*	7	2	0.999
* * *MTOR*	13	3	0.294
* * *CAMK2A*	10	4	0.999
* * *EEF2*	4	1	0.008
* * *HOMER1*	20	3	0.999
* * *GRIA1*	8	6	0.479
* * *EEF2K*	17	4	0.999
* * *HOMER2*	34	5	0.155
* * *GRIA4*	13	2	0.220
* * *GRM5*	31	2	0.485
* * *ARC*	3	1	0.999
			
*Pathway-based set*			
*** *** *mGluR*–*eEf2*–AMPAR	174	5	0.089

Abbreviations: DNHS, Detroit Neighborhood Health Study; GTP, Grady Trauma Project; MTOR, mechanistic target of rapamycin; SNP, single-nucleotide polymorphism.

Analyses conducted only on drinkers who self-identified as African American; gene-based and pathway-based tests were conducted in PLINK using set-based analyses with empirical *P*-values derived from 100 000 permutations. Each analysis was corrected for the number of independent signals (that is, linkage disequilibrium (LD) blocks) within that gene set. Pathway-based tests included all available SNPs within each of the genes included in the mGluR–eEf2–AMPAR pathway, corrected for the number of independent signals (that is, LD blocks) within that pathway set.

**Table 2:** CpG sites available within 100 kb upstream and downstream of eukaryotic translation elongation factor 2 (*EEF2*) in the DNHS

*CpG site*	*Chromosome: BP location*	*eEF2 location*	*Relation to CpG island*	*Regulatory feature*
cg11272616	19: 3 977 495–3978160	Body	South Shore	—
cg11477110	19: 3 984 962–3 985 722	TSS-200	CpG Island	Promoter associated
**cg12255298**	**19: 3** **976** **193**–**3** **976** **193**	**3′-UTR**	**North Shore**	—
cg13634151	19: 3 984 962–3 985 722	TSS-1500	South Shore	Promoter associated
cg16142977	19: 3 984 962–3 985 722	TSS-1500	CpG Island	—
cg17715482	19: 3 977 495–3 978 160	Body	South Shore	—
cg17902989	19: 3 984 962–3 985 722	TSS-200	CpG Island	Promoter associated
cg18064655	19: 3 984 962–3 985 722	5′-UTR	CpG Island	—

Abbreviations: DNHS, Detroit Neighborhood Health Study; TSS, transcription start site; UTR, untranslated region.

Only cg12255298 (in bold) is significantly associated with alcohol consumption in the DNHS (*P*<0.05).

**Table 3:** Key demographic characteristics of the DNHS and GTP genotypic samples

*Key variable*	*Frequency/mean (s.d.)*		
	*DNHS genetic sample*	*DNHS methylation subsample*	*GTP genetic sample*
*N*	778	154	1,034
Female	57.7%	61.2%	71.8%
Age	52.7 (16.4)	50.3 (14.6)	39.5 (13.4)
African American (self-report)	82.5%	91.4%	95.2%
Lifetime drinker	64.3%	30.5%	79.3%
Drinking days per month^a^	5.0 (10.5)	6.3 (15.4)	2.2 (11.5)

Abbreviations: DNHS, Detroit Neighborhood Health Study; GTP, Grady Trauma Project.

a Presented here as non-standardized for ease of interpretation, but analyzed as a *z-*scored standardized continuous measure.
