# Supplementary material for: Frequency of alcohol consumption in humans; the role of metabotropic glutamate receptors and downstream signaling pathways
Source: Transl Psychiatry. 2015 Jun 23;5(6):e586–. doi: 10.1038/tp.2015.70 (PMC4490281; doi:10.1038/tp.2015.70)
Supplement: Supplementary Information [file tp201570x1.doc]

Supplemental Table 1. *MGluR-eEf2-AMPAR* Pathway Predicting Drinking Days Per Month in the DNHS

| **Chr** | **Gene**  **Symbol** | **SNP**  **ID** | **BP**  **Location** | **Major** | **Minor** | **MAF** | **DNHS**  ***p-value*** |
| --- | --- | --- | --- | --- | --- | --- | --- |
| **5** | *CAMK2A* | rs7711562 | 149607616 | G | A | 0.051 | 0.003 |
| **5** | *CAMK2A* | rs887344 | 149529215 | T | C | 0.161 | 0.005 |
| **5** | *CAMK2A* | rs17110944 | 149495590 | T | A | 0.322 | 0.007 |
| **5** | *CAMK2A* | rs919741 | 150232125 | C | T | 0.017 | 0.009 |
| **5** | *CAMK2A* | rs4958469 | 150225553 | G | A | 0.017 | 0.010 |
| **5** | *CAMK2A* | rs2288799 | 149611606 | G | A | 0.169 | 0.012 |
| **5** | *CAMK2A* | rs7714832 | 149655362 | C | T | 0.280 | 0.017 |
| **5** | *CAMK2A* | rs887347 | 149576434 | A | G | 0.042 | 0.022 |
| **5** | *CAMK2A* | rs3776082 | 149524238 | A | G | 0.347 | 0.028 |
| **5** | *CAMK2A* | rs4705430 | 150209046 | G | A | 0.092 | 0.028 |
| **5** | *CAMK2A* | rs919740 | 149646042 | G | A | 0.102 | 0.034 |
| **5** | *CAMK2A* | rs2053053 | 149589586 | G | A | 0.102 | 0.038 |
| **5** | *CAMK2A* | rs2241695 | 149583017 | C | T | 0.136 | 0.040 |
| **6** | *GRM1* | **rs2235875**** | 146762659 | A | C | 0.297 | <0.001 |
| **6** | *GRM1* | rs362848 | 146361349 | G | A | 0.131 | 0.003 |
| **6** | *GRM1* | rs2024589 | 146785596 | C | A | 0.161 | 0.005 |
| **6** | *GRM1* | rs10484925 | 146379240 | T | C | 0.025 | 0.009 |
| **6** | *GRM1* | rs9485078 | 146731953 | C | T | 0.432 | 0.009 |
| **6** | *GRM1* | rs1033534 | 146770909 | G | A | -- | 0.009 |
| **6** | *GRM1* | rs4272232 | 146605615 | C | T | -- | 0.009 |
| **6** | *GRM1* | rs2777475 | 146346296 | A | G | 0.169 | 0.014 |
| **6** | *GRM1* | rs1322210 | 146807439 | C | T | 0.441 | 0.028 |
| **6** | *GRM1* | rs7769505 | 146783226 | G | A | 0.153 | 0.030 |
| **6** | *GRM1* | rs362852 | 146754454 | C | T | 0.492 | 0.033 |
| **6** | *GRM1* | rs362856 | 146761576 | C | A | 0.483 | 0.036 |
| **6** | *GRM1* | rs3804300 | 146775483 | C | T | 0.051 | 0.042 |
| **6** | *GRM1* | rs960385 | 146412767 | G | A | 0.161 | 0.058 |
| **11** | *GRM5* | rs308787** | 87925352 | T | C | 0.347 | <0.001 |
| **11** | *GRM5* | rs11020526** | 87959264 | T | C | 0.415 | <0.001 |
| **11** | *GRM5* | rs7931721** | 89046608 | T | C | 0.017 | <0.001 |
| **11** | *GRM5* | rs495695** | 89061012 | C | A | 0.092 | <0.001 |
| **11** | *GRM5* | rs10831110 | 87963188 | A | G | 0.314 | 0.007 |
| **11** | *GRM5* | rs598758 | 88274783 | T | C | 0.322 | 0.007 |
| **11** | *GRM5* | rs477424 | 88441929 | A | G | 0.475 | 0.007 |
| **11** | *GRM5* | rs308794 | 87922695 | T | C | 0.068 | 0.008 |
| **11** | *GRM5* | rs11018434 | 88405427 | G | A | 0.110 | 0.008 |
| **11** | *GRM5* | rs12807540 | 88219279 | C | T | 0.500 | 0.009 |
| **11** | *GRM5* | rs1353802 | 88070800 | T | C | 0.475 | 0.011 |
| **11** | *GRM5* | rs11607200 | 88524762 | T | C | 0.061 | 0.012 |
| **11** | *GRM5* | rs6483387 | 88079083 | A | G | 0.432 | 0.012 |
| **11** | *GRM5* | rs515570 | 88337621 | G | T | 0.127 | 0.012 |
| **11** | *GRM5* | rs178244 | 87908693 | A | G | 0.212 | 0.013 |
| **11** | *GRM5* | rs7120151 | 89007211 | G | A | 0.208 | 0.013 |
| **11** | *GRM5* | rs961690 | 88074902 | G | A | 0.203 | 0.014 |
| **11** | *GRM5* | **rs10501667** | 88070572 | A | G | 0.042 | 0.016 |
| **11** | *GRM5* | rs1499041 | 87904955 | T | C | 0.398 | 0.016 |
| **11** | *GRM5* | rs538480 | 88035811 | C | T | 0.229 | 0.016 |
| **11** | *GRM5* | rs11021655 | 88890822 | C | A | 0.475 | 0.021 |
| **11** | *GRM5* | rs11823371 | 87921695 | A | G | 0.144 | 0.022 |
| **11** | *GRM5* | rs7121922 | 88398249 | A | C | 0.314 | 0.022 |
| **11** | *GRM5* | rs7396702 | 88020199 | C | G | 0.195 | 0.027 |
| **11** | *GRM5* | rs10501670 | 87938932 | A | G | 0.212 | 0.030 |
| **11** | *GRM5* | rs1892879 | 88430907 | A | C | 0.127 | 0.030 |
| **11** | *GRM5* | rs11021053 | 88090627 | G | A | 0.178 | 0.031 |
| **11** | *GRM5* | rs904493 | 88087865 | C | T | 0.178 | 0.033 |
| **11** | *GRM5* | rs10831183 | 88104997 | T | C | 0.271 | 0.034 |
| **11** | *GRM5* | rs6483414 | 88104488 | T | G | 0.271 | 0.038 |
| **11** | *GRM5* | rs6483430 | 88001883 | G | A | 0.102 | 0.043 |
| **11** | *GRM5* | rs666229 | 88116862 | C | T | 0.492 | 0.045 |
| **11** | *GRM5* | rs12794411 | 88535984 | C | A | 0.123 | 0.047 |
| **11** | *GRM5* | rs7932640 | 88384073 | C | T | 0.347 | 0.047 |
| **11** | *GRM5* | rs6483413 | 88116810 | G | A | 0.102 | 0.048 |
| **11** | *GRM5* | rs308763 | 87929008 | A | G | 0.364 | 0.053 |
| **11** | *GRM5* | rs10741523 | 88160044 | C | T | 0.254 | 0.053 |
| **11** | *GRM5* | rs10830204 | 88427192 | G | A | 0.119 | 0.053 |
| **19** | *EEF2* | rs10420087 | 3902840 | G | A | 0.076 | 0.002 |
| **19** | *EEF2* | rs2289863 | 3979783 | C | T | 0.068 | 0.011 |
| **19** | *EEF2* | **rs3170368**** | 3927321 | G | T | 0.415 | 0.001 |
| **19** | *EEF2* | rs10418537 | 3937003 | G | A | 0.314 | 0.025 |
| **5** | *HOMER1* | rs10075154 | 78664650 | C | T | 0.217 | 0.192 |
| **5** | *HOMER1* | rs2290638 | 78668358 | A | C | 0.118 | 0.368 |
| **5** | *HOMER1* | rs2290637 | 78668550 | C | C | 0.933 | 0.552 |
| **5** | *HOMER1* | rs13360983 | 78685117 | G | T | 0.907 | 0.639 |
| **5** | *HOMER1* | rs6892318 | 78691033 | C | A | 0.978 | 0.070 |
| **5** | *HOMER1* | rs6868393 | 78691697 | G | A | 0.425 | 0.234 |
| **5** | *HOMER1* | rs10038376 | 78694007 | T | C | 0.889 | 0.206 |
| **5** | *HOMER1* | rs6866124 | 78703587 | C | A | 0.903 | 0.389 |
| **5** | *HOMER1* | rs4455546 | 78710606 | A | G | 0.858 | 0.581 |
| **5** | *HOMER1* | rs7708478 | 78719016 | T | C | 0.863 | 0.098 |
| **5** | *HOMER1* | rs7705514 | 78721444 | T | C | 0.580 | 0.353 |
| **5** | *HOMER1* | rs7719054 | 78728923 | C | T | 0.942 | 0.430 |
| **5** | *HOMER1* | rs4292433 | 78729889 | T | C | 0.597 | 0.509 |
| **5** | *HOMER1* | **rs6453450** | 78738510 | C | T | 0.659 | 0.388 |
| **5** | *HOMER1* | rs10039490 | 78740930 | C | T | 0.906 | 0.278 |
| **5** | *HOMER1* | rs12514775 | 78744395 | C | A | 0.504 | 0.183 |
| **5** | *HOMER1* | rs6859667 | 78745042 | A | G | 0.938 | 0.223 |
| **5** | *HOMER1* | **rs6874510** | 78750724 | T | C | 0.629 | 0.263 |
| **5** | *HOMER1* | rs4637531 | 78753539 | C | T | 0.920 | 0.015 |
| **5** | *HOMER1* | rs9293782 | 78759672 | A | C | 0.741 | 0.679 |
| **5** | *HOMER1* | rs12522716 | 78761776 | T | C | 0.920 | 0.472 |
| **5** | *HOMER1* | rs4323213 | 78763345 | A | C | 0.761 | 0.242 |
| **5** | *HOMER1* | rs9293785 | 78769369 | C | T | -- | 0.957 |
| **5** | *HOMER1* | rs10474588 | 78772071 | T | C | 0.743 | 0.091 |
| **5** | *HOMER1* | rs6867782 | 78773959 | C | T | 0.713 | 0.206 |
| **5** | *HOMER1* | rs6868100 | 78774132 | A | C | 0.945 | 0.207 |
| **5** | *HOMER1* | rs10070879 | 78775086 | A | G | 0.767 | 0.435 |
| **5** | *HOMER1* | rs10942889 | 78784281 | A | G | 0.767 | 0.295 |
| **5** | *HOMER1* | rs10057812 | 78796337 | A | G | 0.781 | 0.714 |
| **5** | *HOMER1* | rs4704559 | 78812909 | T | G | 0.881 | 0.535 |
| **5** | *HOMER1* | rs4704560 | 78813750 | T | C | 0.790 | 0.174 |
| **15** | *HOMER2* | rs4779069 | 83505262 | C | T | 0.960 | 0.174 |
| **15** | *HOMER2* | rs2046071 | 83507051 | A | G | 0.522 | 0.413 |
| **15** | *HOMER2* | rs17358518 | 83507377 | C | T | 0.957 | 0.569 |
| **15** | *HOMER2* | rs7174089 | 83514473 | T | C | 0.544 | 0.273 |
| **15** | *HOMER2* | rs899747 | 83516483 | C | T | 0.800 | 0.256 |
| **15** | *HOMER2* | rs955620 | 83516855 | T | C | 0.624 | 0.128 |
| **15** | *HOMER2* | rs1256430 | 83519472 | A | G | 0.935 | 0.859 |
| **15** | *HOMER2* | rs3784377 | 83520228 | G | A | 0.804 | 0.782 |
| **15** | *HOMER2* | rs1256429 | 83521036 | C | T | 0.960 | 0.511 |
| **15** | *HOMER2* | rs1256428 | 83521818 | C | T | 0.609 | 0.981 |
| **15** | *HOMER2* | rs2306428 | 83523387 | T | C | 0.845 | 0.529 |
| **15** | *HOMER2* | rs7175005 | 83523424 | T | C | 0.863 | 0.894 |
| **15** | *HOMER2* | rs1256424 | 83524257 | C | A | 0.761 | 0.334 |
| **15** | *HOMER2* | rs11856299 | 83534421 | T | C | 0.685 | 0.022 |
| **15** | *HOMER2* | rs1256439 | 83538911 | T | C | 0.689 | 0.699 |
| **15** | *HOMER2* | rs17158194 | 83544584 | C | T | 0.783 | 0.357 |
| **15** | *HOMER2* | rs2667384 | 83547026 | C | T | 0.739 | 0.230 |
| **15** | *HOMER2* | rs1588546 | 83547269 | C | A | 0.583 | 0.530 |
| **15** | *HOMER2* | rs17359494 | 83549287 | C | T | 0.913 | 0.093 |
| **15** | *HOMER2* | rs2667385 | 83551313 | G | A | 0.674 | 0.104 |
| **15** | *HOMER2* | rs17158168 | 83565711 | A | C | 0.761 | 0.610 |
| **15** | *HOMER2* | rs12913501 | 83581187 | T | C | -- | 0.950 |
| **15** | *HOMER2* | rs4843146 | 83584170 | T | C | 0.930 | 0.453 |
| **15** | *HOMER2* | rs872598 | 83584579 | A | C | 0.605 | 0.878 |
| **15** | *HOMER2* | rs869498 | 83585402 | T | C | 0.942 | 0.127 |
| **15** | *HOMER2* | rs11259960 | 83588703 | A | G | 0.717 | 0.056 |
| **15** | *HOMER2* | rs12148275 | 83588895 | A | G | 0.804 | 0.638 |
| **15** | *HOMER2* | rs12443081 | 83603563 | A | G | -- | 0.801 |
| **15** | *HOMER2* | rs2061947 | 83606232 | T | C | 0.845 | 0.657 |
| **15** | *HOMER2* | rs7170046 | 83611095 | T | C | 0.978 | 0.288 |
| **15** | *HOMER2* | rs1002881 | 83615274 | A | G | 0.912 | 0.505 |
| **15** | *HOMER2* | rs11857990 | 83623423 | G | A | 0.612 | 0.255 |
| **15** | *HOMER2* | rs4842843 | 83632164 | T | C | 0.891 | 0.440 |
| **15** | *HOMER2* | rs7171145 | 83638814 | C | T | 0.938 | 0.399 |
| **15** | *HOMER2* | rs8028887 | 83646105 | T | C | 0.872 | 0.178 |
| **15** | *HOMER2* | rs6603038 | 83646331 | T | C | 0.786 | 0.660 |
| **15** | *HOMER2* | rs11854758 | 83648780 | C | T | 0.891 | 0.211 |
| **15** | *HOMER2* | rs2167602 | 83649292 | G | A | 0.765 | 0.109 |
| **1** | *MTOR* | rs2536 | 11166713 | C | T | 0.891 | 0.932 |
| **1** | *MTOR* | rs2275525 | 11169676 | T | C | 0.790 | 0.935 |
| **1** | *MTOR* | rs6701524 | 11198502 | G | A | 0.458 | 0.408 |
| **1** | *MTOR* | rs1057079 | 11205058 | T | C | 0.826 | 0.707 |
| **1** | *MTOR* | rs4845853 | 11243470 | G | A | 0.967 | 0.727 |
| **1** | *MTOR* | rs7540001 | 11243833 | G | A | 0.876 | 0.008 |
| **1** | *MTOR* | rs12122605 | 11248020 | T | C | 0.786 | 0.809 |
| **1** | *MTOR* | rs3806317 | 11248216 | G | A | 0.893 | 0.782 |
| **1** | *MTOR* | rs2076658 | 11255008 | C | T | 0.896 | 0.932 |
| **1** | *MTOR* | rs28991014 | 11255238 | G | A | -- | 0.151 |
| **1** | *MTOR* | rs12124598 | 11255431 | T | C | 0.896 | 0.932 |
| **1** | *MTOR* | rs17036508 | 11256034 | C | T | 0.812 | 0.828 |
| **1** | *MTOR* | rs2076655 | 11289161 | A | G | 0.833 | 0.582 |
| **1** | *MTOR* | rs7525957 | 11318236 | C | T | 0.173 | 0.571 |
| **1** | *MTOR* | rs1074078 | 11326788 | T | C | 0.881 | 0.618 |
| **16** | *EEF2K* | rs9925881 | 22216326 | A | G | 0.770 | 0.621 |
| **16** | *EEF2K* | rs11643979 | 22219385 | C | T | 0.978 | 0.296 |
| **16** | *EEF2K* | rs929849 | 22221486 | C | T | 0.863 | 0.081 |
| **16** | *EEF2K* | rs6497580 | 22232426 | G | T | 0.818 | 0.667 |
| **16** | *EEF2K* | rs17841291 | 22240252 | C | T | 0.848 | 0.038 |
| **16** | *EEF2K* | rs4783449 | 22242268 | C | T | 0.580 | 0.136 |
| **16** | *EEF2K* | rs11074507 | 22250569 | A | G | 0.792 | 0.284 |
| **16** | *EEF2K* | rs2316458 | 22251872 | A | G | 0.425 | 0.957 |
| **16** | *EEF2K* | rs2303188 | 22255898 | T | C | 0.833 | 0.778 |
| **16** | *EEF2K* | rs4783451 | 22256193 | C | T | 0.996 | 0.222 |
| **16** | *EEF2K* | rs11639903 | 22257277 | G | A | 0.420 | 0.966 |
| **16** | *EEF2K* | rs2303186 | 22262514 | C | A | 0.500 | 0.681 |
| **16** | *EEF2K* | rs11074509 | 22266403 | A | G | 0.897 | 0.328 |
| **16** | *EEF2K* | rs11640501 | 22268526 | C | A | 0.650 | 0.406 |
| **16** | *EEF2K* | rs8059570 | 22282150 | A | G | 0.587 | 0.806 |
| **16** | *EEF2K* | rs9889102 | 22284842 | A | G | 0.725 | 0.268 |
| **16** | *EEF2K* | rs2873168 | 22289598 | C | A | -- | 0.644 |
| **16** | *EEF2K* | rs11646610 | 22294923 | T | C | 0.616 | 0.311 |
| **16** | *EEF2K* | rs2290829 | 22296486 | A | C | 0.413 | 0.630 |
| **4** | *EIF4E* | rs7669736 | 99788605 | G | T | 0.690 | 0.901 |
| **4** | *EIF4E* | rs10032966 | 99789979 | T | C | 0.478 | 0.375 |
| **4** | *EIF4E* | rs10433891 | 99800310 | G | A | 0.639 | 0.632 |
| **4** | *EIF4E* | rs994636 | 99808872 | C | T | 0.769 | 0.315 |
| **4** | *EIF4E* | rs17570252** | 99822905 | C | T | 0.996 | <0.001 |
| **4** | *EIF4E* | **rs6834230** | 99847876 | T | C | 0.730 | 0.041 |
| **4** | *EIF4E* | rs11723037 | 99850126 | A | G | 0.942 | 0.620 |
| **4** | *EIF4E* | rs13107892 | 99852395 | C | T | 0.900 | 0.001 |
| **5** | *GRIA1* | rs578772 | 152867348 | T | C | 0.886 | 0.456 |
| **5** | *GRIA1* | rs548294 | 152868437 | T | C | 0.688 | 0.685 |
| **5** | *GRIA1* | rs3792766 | 152869624 | G | A | 0.934 | 0.533 |
| **5** | *GRIA1* | **rs3828595** | 152871721 | T | G | 0.929 | 0.748 |
| **5** | *GRIA1* | rs4145160 | 152874549 | A | G | 0.891 | 0.626 |
| **5** | *GRIA1* | rs509555 | 152876121 | G | A | 0.544 | 0.613 |
| **5** | *GRIA1* | **rs1428920** | 152877008 | T | C | 0.677 | 0.445 |
| **5** | *GRIA1* | rs13164993 | 152877509 | T | G | 0.978 | 0.984 |
| **5** | *GRIA1* | rs540375 | 152878727 | G | A | 0.965 | 0.108 |
| **11** | *GRIA4* | rs17104715 | 105791235 | T | C | 0.630 | 0.011 |
| **11** | *GRIA4* | rs599980 | 105719344 | G | A | 0.819 | 0.021 |
| **11** | *GRIA4* | rs675091 | 105853107 | G | A | 0.804 | 0.042 |
| **11** | *GRIA4* | rs11226805 | 105482024 | T | C | 0.457 | 0.658 |
| **11** | *GRIA4* | rs1562221 | 105483629 | T | C | 0.571 | 0.116 |
| **11** | *GRIA4* | rs2166318 | 105483661 | T | G | 0.783 | 0.727 |
| **11** | *GRIA4* | rs7949759 | 105484844 | C | T | 0.522 | 0.107 |
| **11** | *GRIA4* | rs10750731 | 105494105 | A | C | 0.580 | 0.812 |
| **11** | *GRIA4* | rs1445604 | 105498095 | C | T | 0.881 | 0.950 |
| **11** | *GRIA4* | **rs474158**** | 105342254 | C | T | 0.553 | <0.001 |
| **11** | *GRIA4* | rs10791764 | 105514964 | G | A | -- | 0.164 |
| **11** | *GRIA4* | rs12421796 | 105522961 | G | A | 0.935 | 0.290 |
| **11** | *GRIA4* | rs11226820 | 105529933 | A | G | 0.929 | 0.321 |
| **8** | *ARC* | rs10103454 | 143688896 | A | C | 0.106 | 0.394 |
| **8** | *ARC* | rs10110456 | 143690833 | A | G | 0.112 | 0.114 |
| **8** | *ARC* | rs10097505 | 143694184 | A | G | 0.335 | 0.112 |

*Note: Bolded Variants Replicated in the Grady Trauma Project (Phenotype: frequency of alcohol consumption and related adverse behaviors/consequences); **Indicates Variants that Withstand a Bonferroni Correction for Multiple Testing; Post-hoc analyses of individual SNPs included in the MGluR-eEf2-AMPAR pathway were conducted to provide further information on each individual variant. These analyses were conducted in PLINK using linear regression for a quantitative phenotype.*
